# Sirt1 Activity in the Brain: Simultaneous Effects on Energy Homeostasis and Reproduction

**DOI:** 10.3390/ijerph18031243

**Published:** 2021-01-30

**Authors:** Stefania D’Angelo, Elena Mele, Federico Di Filippo, Andrea Viggiano, Rosaria Meccariello

**Affiliations:** 1 Department of Movement Sciences and Wellbeing, University of Naples Parthenope, 80133 Napoli, Italy; stefania.dangelo@uniparthenope.it (S.D.); elena.mele@collaboratore.uniparthenope.it (E.M.); 2 Deptartment of Medicine, Surgery and Dentistry “Scuola Medica Salernitana”, via Allende, 84081 Baronissi (SA), Italy; federicodifilippo@hotmail.it (F.D.F.); aviggiano@unisa.it (A.V.)

**Keywords:** Sirt1, brain, energy homeostasis, reproduction, kisspeptin, polyphenols

## Abstract

Diet deeply impacts brain functions like synaptic plasticity and cognitive processes, neuroendocrine functions, reproduction and behaviour, with detrimental or protective effects on neuronal physiology and therefore consequences for health. In this respect, the activity of metabolic sensors within the brain is critical for the maintenance of health status and represents a possible therapeutic target for some diseases. This review summarizes the main activity of Sirtuin1 (Sirt1), a metabolic sensor within the brain with a focus on the link between the central control of energy homeostasis and reproduction. The possible modulation of Sirt1 by natural phytochemical compounds like polyphenols is also discussed.

## 1. Introduction

The World Health Organization (WHO) recognizes the double burden of malnutrition, including undernutrition and overweight, as a serious public health challenge facing many countries worldwide. In recent years, nutrition-related pathologies emerged as an epidemiologic pandemic: among them, obesity has the greatest incidence and prevalence, gaining the meaningful definition of “globesity” [1] and affecting more than 33% of the USA population [2] with an astonishing increasing rate of almost 50% over the past 35 years [3]. Its clinical relevance for the development of comorbidities was studied for the first time in the famous Framingham experiment [4], which begun in the late 1940s, that showed that obesity is an independent risk factor for cardiovascular diseases and mortality in patients with a BMI > 30 [5].

Similarly, a remarkable deficit in fertility rate has been reported in developing countries [6] and accordingly to WHO [7] “infertility affects millions of people of reproductive age worldwide and has an impact on their families and communities. Estimates suggest that between 48 million couples and 186 million individuals live with infertility globally”. Several environmental factors such as nutritional status, lifestyle, exposure to endocrine disruptors, and others can affect gametogenesis and gamete quality in both sexes, pregnancy and embryo development [8,9,10,11,12].

The metabolic status strictly affects tissue homeostasis and reproduction. Diet represents an epigenetic modulator of gene expression, being capable of modulating gene transcription without any effect on gene sequence with mechanisms involving DNA methylation, chromatin remodeling and the production of non-coding RNA [13,14,15,16,17,18,19,20]. Epigenetic modulation of gene expression may lead to disease in later life and trans-generational effects in the offspring [21,22,23,24].

Brain function strongly depends on energy availability and diet deeply impacts brain function with detrimental or protective effects on neuronal physiology and therefore consequences for health [25,26,27]. Impairment of synaptic plasticity, cognitive processes and neuroendocrine functions, are the main consequences of a high fat diet [25,26] and neuronal damage and are notably associated to disease load.

Specific brain regions of the hypothalamus capture metabolic information and modulate physiological mechanisms for energy balance [25]. In addition, reproduction depends on the hypothalamic release of the gonadotropin releasing hormone (GnRH), which sustains sex steroid biosynthesis and gametogenesis upstream, with several environmental cues, including those related to the metabolic status, capable of modulating the activity of the hypothalamus–pituitary–gonad (HPG) axis [25]. Therefore, understanding the neuro (endocrine) background for energy homeostasis could lead to improvement of therapeutic strategies.

In these respects, the activity of metabolic sensors within the brain is critical for the maintenance of health status and represents a possible therapeutic target for some diseases. This review summarizes the main activity of the metabolic sensors Sirtuin 1 (Sirt1) in the brain with a focus on link between the central control of energy homeostasis and reproduction. The possible modulation of Sirt1 by natural phytochemical compounds like polyphenols is also discussed.

## 2. Sirt1

The sirtuin family comprises seven proteins (Sirt1–7 in mammals), among which Sirt1, the mammalian homologue of the yeast Sir2 (silent information regulator 2 homologue 1), is the most conserved and characterized [28]. Sirt1 is a nicotinamide adenine dinucleotide (NAD^+^)-dependent class III deacetylase acting as a cellular energy sensor and is linked with successful aging, calorie restriction (CR), metabolism, cellular differentiation and apoptosis [29]. Sirt1 is expressed in many tissues, from brain to liver or gonads [30,31,32,33]. Its canonical localization is within the nucleus, but, differently from other members of the sirtuin family (Table 1 for details), Sirt1 has the ability to translocate to the cytoplasm in response to physiological stimuli or disease status.

**Figure 1:**
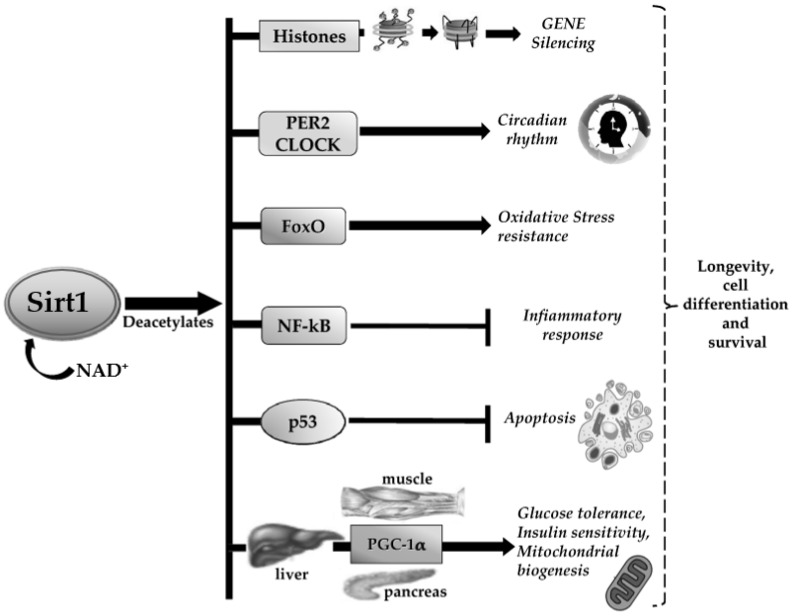
Schematic representation of the main activities of Sirt1 in biological tissues.

Notably, Sirt1 acts on many epigenetic and non-epigenetic targets. It modulates the biological activity of target proteins thought the removal of functional acetyl groups; its main molecular targets are histone proteins with consequent remodelling of chromatin architecture, transcription factors such as tumor protein P53 (TP53 or P53), the forkhead transcription factor 1 (FoxO1) and co-factors like peroxisome proliferator-activated receptor gamma (PPARγ) coactivator 1-alpha (PGC-1α) [34]. The downstream Sirt1-related pathways include cell differentiation and survival, inflammation, apoptosis and autophagy, mitochondrial biogenesis, lipid/glucose homeostasis through the transcriptional activation/repression of genes involved in metabolism, aging, cell death or circadian clock, among others [35,36,37], hence the assumption of Sirt1 as a “longevity gene”. Figure 1 summarizes the main activities of Sirt1 in biological tissues.

The Sirt1 deacethylase activity depends on the NAD^+^/NADH ratio, requiring NAD^+^ availability and therefore active metabolism. For this reason, Sirt1 is considered a nutrient/redox sensor and its activity in peripheral organs like liver or adipose tissue is well known and has been excellently reviewed [38].

In general, in adipose tissue, Sirt1 promotes lipolysis, decreasing fat storage, and exerts protective action against obesity-induced inflammation [39,40,41]; in the liver it affects fatty acid metabolism and it increases oxidative metabolism [42,43]; in the pancreas it improves glucose tolerance, regulating insulin secretion [44,45]. Fasting increases Sirt1 to gain fatty acid oxidation and gluconeogenesis, thus suppressing adipogenesis, and insulin secretion and action, whereas overnutrition suppresses its activity in vertebrates, including humans (see [38] for review).

Interestingly, Sirt1 expression is highly susceptible to dietary manipulation and environmental factors like the exposure to toxicants or endocrine disruptors [38,46], thus representing a key factor in the preservation of tissue physiology and a possible therapeutic target for metabolic disorders and related diseases.

## 3. Sirt1 Activity in the Brain

**Figure 2:**
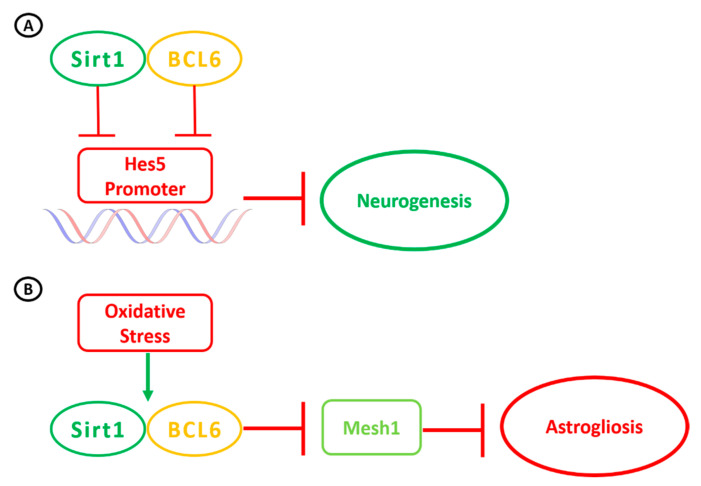
Key role of Sirt1 in neurogenesis and astrogliosis under different circumstances. (**A**) Repression of *Hes5* genes by Bcl6 promotes neurogenesis in NSCs. Bcl6 recruits SIRT1 to inhibit *Hes5* promoter and its transcription, resulting in activation of neurogenetic pathways. (**B**) Oxidative stress upregulates Sirt1, promoting its pairing with BCL6: thus, the complex inhibits neurogenetic factor Mash1—through the Hes1 pathway—inducing astrogliosis.

Sirt1 has been reported to be largely expressed in both neurons and glial cells in vivo, and in microglia, neuronal stem cells and astrocytes in vitro [47]. Within the hypothalamus, Sirt1 mRNA is highly expressed in the arcuate, ventromedial, dorsomedial and paraventricular nuclei. Its activity within the brain has been linked to brain aging, neuroprotection against oxidative stress, neuroinflammation and ischemic injury, central control of energy homeostasis, modulation of circadian clock, and neuroendocrine functions [35,48,49,50,51,52,53,54,55,56,57,58,59,60,61,62]. Sirt1 expression regulates both the fate of neural stem cells (NSCs) during development and the activity of neurons through several pleomorphic pathways [63]. Under different circumstances and according to its non-uniform distribution in different brain regions, both Sirt1 activation and silencing may lead to neurotrophic changes, resulting in cellular differentiation, increased neurogenesis and neuritogenesis or reduced inflammatory state. Its activity, depending on the tissue and cellular redox state, induces differentiation of NSCs towards the astroglia lineage by reducing the levels of the pro-neuronal transcription factor Mash-1 [64]: in fact, DNA glycosylase knock-out mice accumulate mitochondrial DNA damage in differentiating neural cells, which shift to astrocytic lineage because of an increased NAD^+^/NADH ratio eliciting Sirt1 over-activation [65]. On the other hand, Sirt1 inhibition promotes neuronal differentiation [64] in the subventricular zone and in the hippocampus [66]: its silencing increases the level of acetylated Pax3, inducing a decrease in Hes-1 (that helps maintaining stem cell) and an increase in Neurog2 (with neuronal-differentiation properties) activities, which overall promotes neurogenesis [67]. Conversely, in different contexts, pro-neurogenic Bcl6 has been shown to promote neurogenesis through Sirt1 recruitment and activation, with the silencing of Hes-5 in the Notch signaling pathway [68] (Figure 2).

**Figure 3:**
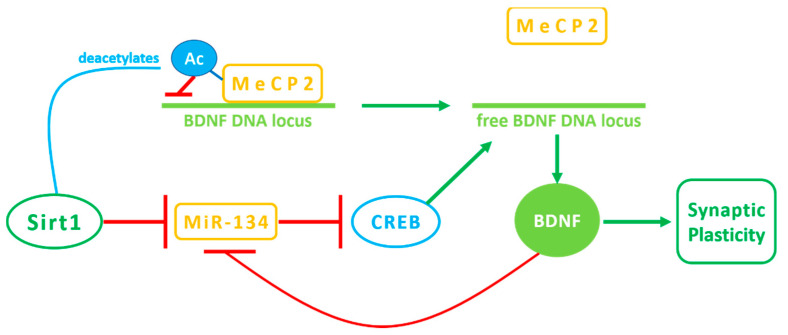
Sirt1 modulation of synaptic plasticity. Sirt1 downregulates the expression of miR-134, promoting the activation of the CREB-BDNF axis. BDNF transcription could also be intensified through MeCP2 deacetylation by Sirt1.

Other reports have indicated that manipulations of Sirt1 expression or activity enhances nerve growth factor-induced neuritogenesis, the outgrowth of axons and dendrites [69]. Regeneration of peripheral axons after injury is Sirt1-dependent and expression of Sirt1 in hippocampal neurons enhances dendritic arbor complexity, especially in CA1 neurons, whose dendritic spine density is reduced proportionally to Sirt1 depletion in some forms of neurodegeneration [63,70]. Moreover, Sirt1 protein activities contribute to synaptic plasticity and neuroprotection, slowing down brain aging [71,72]. The Sirt1-deficient hippocampus has decreased levels of key neurotrophins regulating synaptic functions, such as synaptophysin and neurotrophic factor (BDNF). This can be also explained by a reduction in cAMP response element-binding protein (CREB) binding to several neurotrophic promoters, as the levels of CREB protein in Sirt1-deficient brain decreased due to both an upregulation of miR-134, which Sirt1 normally suppresses, and a downregulation of methyl-CpG bindingprotein2 (MeCP2) deacetylation, which Sirt1 normally promotes [73] (Figure 3).

As a consequence, the dysregulation of Sirt1 activity within the brain or its modulation by environmental factors like diet, deeply impacts brain function with heavy consequences on health status. Table 2 summarizes the main activities of Sirt1 within the brain with the exception of that related to energy homeostasis and reproduction, which are described in Section 4.

Taken together, dysfunction in specific neuronal populations within the hypothalamus is related to physiological or pathological brain aging. The dysregulation of nutrient sensing, impaired neuronal communication network, NSC exhaustion, impaired repair mechanisms and alterations in the epigenetic machinery have age-related consequences on the decline in energy metabolism, hormone regulation, circadian rhythm and reproduction [74]. In this respect, Sirt1, mammalian target of rapamycin (mTOR), AMP-activated kinase (AMPK), and nuclear factor kappa-light-chain-enhancer of activated B cells (NF-kB) are critical factors/pathways.

Moreover, microglia is critical for neuroinflammatory processes related to aging brain, where inflammation of microglia is related to the loss of NSCs in the mediobasal hypothalamus [75]. To date, ectopic activation of Sirt1 in the brain and dominantly acting NF-κB inhibition in hypothalamic microglia/NSCs extend life span in mice [75]. However, the current hypothesis does not consider NCSs as a mere reservoir for neuronal differentiation, but also a physiological effector capable of providing modulators like non-coding RNAs in exosomes that oppose aging-associated neurological and skeletomuscular dysfunction [75].

## 4. Sirt1 and the Relationship between the Central Control of Energy Homeostasis and Reproduction

The hypothalamus is a control centre for energy homeostasis and reproduction within the brain [76,77]. It maintains energy homeostasis by catching and integrating environmental cues, including nutrient availability, and correlates the (neuro)endocrine system to physiological functions, through the communication with “second level” neurons located within the hypothalamus itself or in extra-hypothalamic brain areas. Several peripherally produced “metabolic sensors” like leptin from white adipose tissue, insulin from the pancreas or ghrelin from the gastro-intestinal tract, among the major modulators of energy expenditure and appetite, provide the brain information related to energy status [76,78]. Within the medio-basal hypothalamus, the arcuate nucleus (ARC) contains specific neuronal populations that produce orexigenic and anorexigenic factors that respectively stimulate appetite (e.g., neuropeptide Y (NPY) and agouti related protein (AgRP)) or inhibit appetite (i.e., proopiomelanocortin (POMC) and cocaine-amphetamine regulated transcript (CART)) to adapt food intake in response to nutrient availability.

The hypothalamus also controls reproduction by releasing the hypothalamic gonadotropin releasing hormone (GnRH) [77]. This decapeptide is responsible for the discharge of pituitary gonadotropins, which, in turn, sustain the production of sex steroids from the gonads [77]. Energy homeostasis has deep effects on reproduction. Data from clinical studies and from dietary, pharmacological or genetic manipulations in animal models reveal that obesity and under-nutrition cause reproductive disorders, due to the close link between the neuronal networks controlling food intake and the hypothalamus-pituitary-gonad (HPG) axis [25,79]. Kisspeptin, the product of the *KISS1* gene, is the main upstream positive modulator of GnRH secreting neurons and the main intermediate in the communication along the HPG axis [80]. Therefore, it represents a critical factor in the neuronal circuits regulating metabolism and reproduction. Similarly, endocannabinoids, lipid mediators acting on cannabinoid receptors [81], are strongly involved in the control of food intake and gut–brain communications [82] and negatively affect GnRH secretion [83,84].

Within the hypothalamus, SIRT1 is expressed in the POMC and AgRP neurons of the ARC and in the steroidogenic factor 1 (SF1) neurons of the ventromedial hypothalamic nucleus (VMH), suggesting a critical role in the control of energy homeostasis [85]. In particular, Sirt1 activity is involved in the production of hypothalamic peptides that directly or indirectly regulate energy balance, thus representing a molecular switch for the expression of appetite-related inhibiting and appetite inducing neuropeptides [78,85]. For example, POMC is cleaved to produce the bioactive peptide α-melanocyte stimulating hormone (α-MSH) or the stress related corticotropin-releasing hormone (CRH) within the PVN. In such a process Sirt1 operates with direct or FoxO1-mediated effects on the transcription rate of the specific genes (direct transcriptional regulation) or through the regulation of the prohormone convertases, the enzymes involved in the post-translational maturation of pro-hormones (indirect post-transcriptional mechanisms) [78].

Data from knockout mice or pharmacological inhibition or cell specific genetic inactivation of Sirt1 provide evidence that Sirt1 has protective roles against dietary metabolic imbalance [86]. Caloric restriction or fasting specifically increases Sirt1 expression within the hypothalamus [87,88], with a consequent decrease in FoxO1 acetylation and through the regulation of S6K signalling. Accordingly, decreased body weight gain and food intake are the main consequences of the pharmacological inhibition or siRNA mediated knock down of hypothalamic Sirt1 [89]. Moreover, SHU9119, a specific POMC antagonist, fully reversed the effects of Sirt1 depletion in the hypothalamus [89]. Interestingly, the metabolic actions of melanocortin (MCH) are reduced in mice lacking Sirt1, specifically in POMC neurons, revealing that the Sirt1/FoxO1 pathway also regulates the inhibitory effect of MCH on POMC expression, thus mediating MCH-induced feeding, adiposity, and glucose intolerance [90], and confirming the relevance of Sirt1 activity in obesity.

In the hypothalamic POMC neurons, Sirt1 is also involved in the leptin-mediated regulation of metabolism. Leptin, an anorexigenic hormone secreted by adipose tissue, suppresses body weight gain through the activation of molecular pathways requiring the leptin receptor (OBR), the signal transducer and activator of transcription 3 (STAT3) and phosphatidylinositol-4,5-bisphosphate 3-kinase (PI3K) [91]. The genetic impairment of Sirt1 in POMC neurons causes hypersensitivity to diet-induced obesity due to reduced energy expenditure and compromises the remodelling of white adipose tissue [91]. Consistently, Sirt1 overexpression in POMC or AgRP neurons improves leptin sensitivity in mice and reduces food intake [92]. However, the consumption of a high fat diet compromised the aforementioned effects, confirming that the expression of Sirt1 in the hypothalamus depends on NAD^+^ availability [92]. In addition, the physiological function of leptin requires the deacethylasing activity of Sirt1 outside the ARC, in the SF1 neurons of the VMC [86].

Recent studies point out sex specific differences in the outcomes of Sirt1 overexpression/inactivation in mouse neurons or glia cells [93,94], suggesting that females are more sensitive to the metabolic improvements and suppression of reproduction by dietary intervention [93]. In this respect, Sirt1 controls fertility in mice through the regulation of the HPG axis. Sirt1 knock out (KO) mice (*Sirt1^-/-^*) are affected by infertility in both sexes with oocytes and sperm failing to mature [95]. Male mice have meiotic spermatogenesis arrest, high apoptosis rate of pachytene spermatocytes, abnormal maturation of somatic cells (both Leydig and Sertoli cells) and reduced intratesticular testosterone levels. This reproductive phenotype is the consequence of a significant reduction in the hypothalamic expression of GnRH and low gonadotropin discharge (both LH and FSH) in KO mice [96]. Defective migration of GnRH neurons has been identified in Sirt1^-/-^ mice [97]. At the molecular level, the Sirt1 binding and deacetylation of cortactin in parallel to the involvement of fibroblast growth factor 8 and fibroblast growth factor receptor-1 have been discovered [97]. In addition, the downstream GnRH effects such as the stimulation of FSH discharge from the pituitary requires the *miR-132/miR-212* dependent expression of *FSH beta subunit* (*FSHβ*) and involves a Sirt1-FoxO1 pathway in rat pituitary cells and mouse LβT2 gonadotrope cells [98]. In fact, the decrease in Sirt1 deacethylating activity, causes an increased acethylation of FoxO1, a transcriptional repressor of *FSHβ*, that translocates from the nucleus to the cytoplasm to attenuate its repressive effect on *FSHβ* transcription [98]. Taken together, these observations reveal a direct involvement of Sirt1 in the control of the reproductive axis.

Puberty is a maturational critical life stage involving developmental changes and the activation of the HPG axis. The main gatekeepers of this process are kisspeptin neurons and the major modulators are gonadal sex steroids via feedback loops. Notably, loss or gain of function mutations in either *Kiss1* or *Kiss1 receptor* (*Kiss1R*) genes respectively cause hypogonadotropic hypogonadism or precocious puberty in mice and humans [99]. In the hypothalamus, kisspeptin neurons populate the anteroventral periventricular nucleus (AVPV) and the ARC, which are notably involved in the positive and negative feedback loops by sex steroids on GnRH [80]. A subpopulation of neurons termed KNDy located within the ARC and coexpressing kisspeptin (K), neurokinin B (N) and dynorphin A (Dy), positive and negative modulators of GnRH respectively, is a key mediator of pulsatile GnRH secretion [80]. Puberty onset is highly dependent on environmental factors and energy homeostasis, and the expression of *Kiss1* has been reported to be epigenetically modulated [100], with methylation patterns of both *Kiss1r* and *Kiss1* gene promoters changing across puberty [101].

Interestingly, Vazquez and co-workers [102] recently revealed an inverse relationship between *Kiss1* and Sirt1 within the ARC in relationship to puberty onset and nutritional status. In fact, the increase in *Kiss1* expression observed in the hypothalamus at pubertal maturation, parallels a decrease in the content of Sirt1, which in turn is modulated by nutritional status. Accordingly, diet manipulations in animal models affect Sirt1 with consequent *Kiss1* expression at puberty onset [102], thus suggesting an inverse relationship between *Kiss1* and Sirt1 within the ARC [79]. A molecular mechanism has been demonstrated in female rats, revealing that puberty onset requires the epigenetic modulation of the hypothalamic *Kiss1* via Sirt1 activity. In fact, Sirt1 in the ARC contributes to the transcriptional repression of *Kiss1* through the interaction with the Polycomb (PcG) silencing complex, thus causing a repressive histone configuration at the *Kiss1* promoter. At puberty onset, Sirt1 is evicted from the *Kiss1* promoter and *Kiss1* transcription occurs. Therefore, dietary manipulation such as over- or under-nutrition may anticipate or delay puberty onset respectively, through the premature or delayed removal of Sirt1 from the *Kiss1* promoter [79,102].

Another interesting relationship between puberty onset and feeding is represented by the interaction between leptin signalling and Sirt1 expression. Mice lacking leptin (*ob/ob*) or leptin receptor (*db/db*) are both obese and infertile, and leptin administration to *ob/ob* mice normalizes food intake, body weight and hypogonadism [103,104]. This observation suggests that leptin can represent a signal to the hypothalamus about the overall nutritional state [105]. According to this model, the hypothalamus starts (puberty) and promotes gonadal function when the nutritional state is adequate. Sirt1 expression then appears as the intracellular switch activated by leptin in POMC neurons of the ARC nucleus of the hypothalamus, linking leptin levels to GnRH production and gonad stimulation.

However, changes in blood concentration or dysfunction in endocrine pathways of leptin, adiponectin, resistin and kisspeptin could lead to dysregulation of fertility [106]: therefore, chronic impairment of nutritional balance (undernutrition, obesity and diabetes) or alterations in leptin or kisspeptin pathways [107] are risk factors for hypogonadotropic hypogonadism and infertility [108,109].

Taken together, Sirt1 activity within the hypothalamus is critical for the control of energy homeostasis and reproduction and the maintenance of its biological activity may be functional to prevent obesity and reproductive dysfunction.

## 5. Dietary Preservation of Sirt1 Activity: The Role of Polyphenols

As sirtuins are attractive therapeutic targets, considerable effort has been directed towards developing particular sirtuin inhibitors and activators, as tools for studying their function and potentially as treatments for age-related situations [110,111]. The properties of sirtuins can be modulated by diverse activators [110]. Fluctuations in the availability of NAD^+^ via an increment in its biosynthesis or via non-allosteric methods that raise sirtuin levels, such as nicotinamide riboside and nicotinamide mononucleotide, can modulate Sirt1 activity [112]. The so-called sirtuin activating compounds (STACs) are a group of molecules that can increment sirtuin’s actions.

Numerous molecules, comprising natural phytochemical compounds, can modify Sirt1 activity. The most potent molecule reported to date is resveratrol, a polyphenol, but others phytonutrients, such as curcumin, tannins, quercetin, and catechins can also enhance sirtuin’s actions [113]. Natural polyphenols are the largest group of phytochemicals and are potential agents for the prevention and treatment of stress-related oxidative syndromes. Polyphenols are secondary metabolites of plants and represent a vast group of compounds with aromatic ring(s), characterized by the presence of one or more hydroxyl groups with variable structural complexities. The number and characteristics of these phenol structures underlie the unique chemical, physical, and biological properties. There are currently about 8000 diverse polyphenols. Considering the number and arrangement of phenolic rings, these nutraceuticals can be separated into two classes: (1) flavonoids, compraising flavanols, isoflavones, flavanones, flavonones, and anthocyanidins; (2) non-flavonoids, such as phenolic acids (groups of compounds derived from benzoic and hydroxycinnamic acids), stilbenes, and lignans [114].

**Figure 4:**
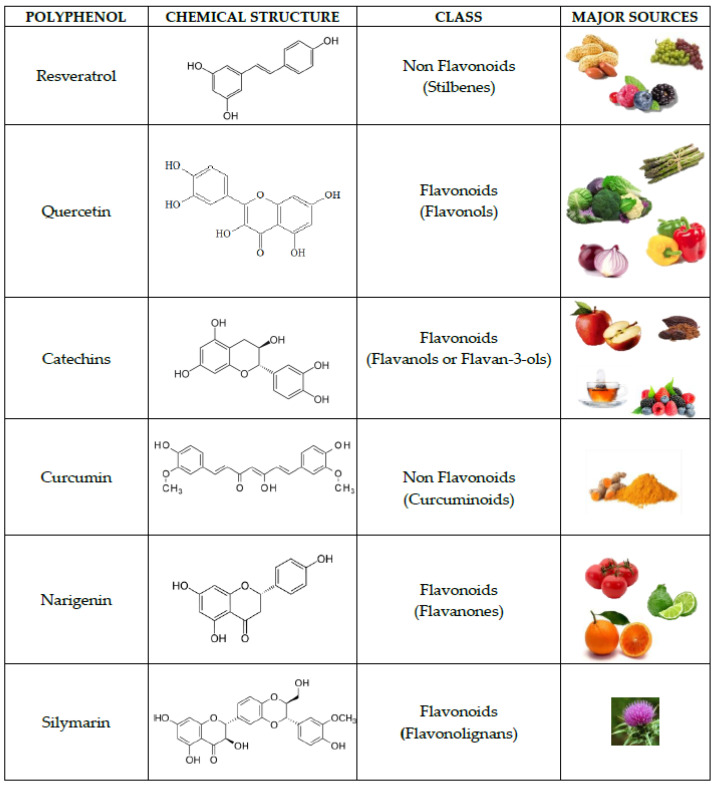
Some polyphenols involved in Sirt1 activity.

They are found in many plants and foods, such as vegetables, fruits, tea and wine, and long-term intake is correlated with health effects. Mediterranean diets are in fact linked to a decreased risk of chronic syndromes due to the intake of red wine and olive oil, which contain high quantities of polyphenols [46,115]. Polyphenols have various biological properties [116,117,118,119] and in particular, they are antioxidants [120,121,122]. A peculiar activity of polyphenols is their ability to cross the blood–brain barrier and to protect brain cells from damage and preserve their crucial function [114]. Polyphenols favor the activation of some anti-aging proteins, like Sirt1, which affects synaptic plasticity and memory. The mechanism inside this set of brain properties can be associated with the anti-inflammatory and antioxidant activities of polyphenols. Polyphenols defend lipids, carbohydrates, proteins, and DNA from oxidative injury, and they also induce augmented levels of antioxidant defense systems such as the superoxide dismutase, glutathione peroxidase, and ascorbic acid [123]. Figure 4 shows some polyphenols involved in Sirt1 activity.

Resveratrol is a polyphenol with numerous properties in neurological disorders. It is found in many plants, including peanuts, grapes, and berries. It has been shown to mimic the actions of caloric restriction, indicating anti-inflammatory and anti-oxidative properties [124,125] and to hinder the progression of many syndromes [126]. Resveratrol has been found to improve brain health through multiple signaling pathway mechanisms through Sirt1. The regulatory mechanisms comprise anti-oxidative, anti-inflammatory, and anti-apoptotic processes and autophagy regulation, as well as increases in cerebral blood flow and enhancements in the plasticity of synaptic pathways [127]. Resveratrol also has anti-inflammatory properties as it suppresses M1 microglia stimulation, which is involved in the start of neurodegeneration, and promotes Th2 responses by increasing anti-inflammatory cytokines and Sirt1 expression. Resveratrol can indirectly stimulate Sirt1 expression and lead to neuro-protection in Alzheimer’s disease [128,129].

Administration of the polyphenols silymarin, quercetin and naringeninin results in restorative actions on cognition and motor coordination in rats. These polyphenols reversed the age-induced deficits in monoaminergic neurotransmitters and amplified Sirt1 levels and reduced NF-κB levels in the hippocampus [130].

Curcumin (an orange yellow component of turmeric or curry) reduces ischemic stroke-induced brain injury via activation of Sirt1 in rats [131].

Catechins are polyphenols present in many dietary foods, plants, fruits as apples, gooseberries, blueberries, grape seeds, strawberries, kiwi, red wine, green tea, beer, cacao liquor, cocoa, and chocolate. Chronic catechin treatments increase hippocampal Sirt1 levels improving cognition in aged rats [132]. Intake of green tea extract or catechins reverted the age-associated decrease of the neuro-inflammation by modulating Sirt1 expression in the hippocampus, recovering levels of Sirt1 proteins in old animals, reaching values similar to those found in young animals.

This result reinforces the important role of Sirt1 as one of the responsible agents in the neuroprotective actions of therapies improving memory. In vivo, the defensive action from these therapies in brain Sirt1 levels seems to be mainly due to a defensive action against an oxidative status. These outcomes propose a general defensive effect of all of these compounds on age-associated brain decline, pointing to a decrease in oxidative damage and neuro-inflammatory status as the leading mechanisms. Therefore, polyphenols can defend Sirt1 enzyme due to their antioxidant effects and, in turn, modulate proteins affected by Sirt1 activity [133].

Numerous reports highlighted that dietary supplementation with polyphenols can defend against cardiovascular, neurodegenerative, metabolic inflammatory diseases and cancer by enhancing Sirt1 deacetylase action [46,127,128,129,130,131,132,133,134,135,136,137,138,139,140]. However, the therapeutic and pharmacological potential of these natural compounds remains to be translated to humans in clinical trials. This is in part due to the lack of knowledge of their mode of action as well as their multiple signaling targets, non-specificity, complex pharmacokinetic effects (e.g., absorption, bioavailability and biotransformation). Furthermore, these polyphenols may act as pre-emptying or prophylactic agents in terms of dietary intake/interventions rather than as therapeutic agents [141].

## 6. Conclusions

The WHO recognizes the double burden of malnutrition, including undernutrition and overweight, as a serious public health challenge facing many countries worldwide. Energy homeostasis conserves energy excess as fat and provides energy for immediate metabolic needs or energy demanding process, like reproduction. Within the hypothalamus, the ARC guarantees tissue homeostasis and physiology expressing numerous neuropeptides including kisspeptin, the main gatekeeper for reproduction. Therefore, chronic impairment of nutritional balance, such as undernutrition, obesity and diabetes, and alterations in leptin or kisspeptin pathways are risk factors for infertility. In this respect, studies aimed at understanding the neuroendocrine background for energy homeostasis and reproduction could lead to improved therapeutic strategies for disease treatment or load.

Sirt1 is a critical metabolic sensor capable of modulating epigenetic and non-epigenetic targets within the brain. In the ARC, it is deeply involved in the control of energy homeostasis and in the diet-dependent modulation of reproduction via the epigenetic modulation of *Kiss1*. Sirt1-sex specific protecting roles against high-fat-induced obesity and metabolic derangements has been reported suggesting that dietary supplementation with natural phytochemical compounds like polyphenols, which are capable of modulating Sirt1 activity, may be useful to preserve Sirt1 activity.

## Figures and Tables

**Table 1:** Cellular localization (X) and additional activities of Sirt1–7.

	Cytoplasm	Nucleus	Mitochondrion	Additional Activities with Respect to NAD+—Dependent Removal of Acetyl Groups from Target Proteins [28]
Sirt1	X	X	-	
Sirt2	X	transient	-	Demyristoylase
Sirt3	-	-	X	
Sirt4	-	-	X	ADP-ribosyltransferase
Sirt5	-	-	X	DemanlonylaseDesuccinylase
Sirt6	-	X	-	ADP-ribosyltransferase
Sirt7	-	X	-	

**Table 2:** Sirt1 activity in the brain.

Activity	Effect	Reference
Aging, Neuroprotection, and Neurodegeneration	Sirt1 is implicated in life span extension in mice that are either calorie restricted or on standard diet.	[48]
	Sirt1 proteinis sensitive to caloric deficit and mediate the beneficial effects of caloric restriction	[49]
	A caloric restriction can activate sirtuins via an increase in NAD+ levels. The Sirt1/mTOR signaling pathways in the brain are involved in the mechanisms of neuroprotection of caloric restriction.	
	Up-regulation of Sirt1 and PGC-1α expression improved learning and memory abilities	[50]
	In Alzheimer’s disease mice models, treadmill exercise inhibited the production of β- amyloid via Sirt1, favoring the non-amyloidogenic pathway of Alzheimer’s disease	[51]
	Decline in serum concentration of Sirt1 in healthy individuals as they age	[52]
	Sirt1 serum concentration declines in patients diagnosed with Alzheimer’s disease and mild cognitive impairment when compared to elderly and young controls	
	In the inducible p25 transgenic mouse, a model of Alzheimer’s disease and tauopathies, enhancement of Sirt1 activity by resveratrol or injection of Sirt1 recombinant lentivirus in the hippocampus resulted in significant protection against neurodegeneration by deacetylation of PGC-1α and P53	[53]
	Protective role of Sirt1 in age-related cognitive decline such as Alzheimer’s disease Parkinson’s disease and Lewybody dementia	[54]
	Microglial Sirt1 deficiency is a causative role in cognitive decline and neurodegeneration	[55]
	Sirt1 (both mRNA and protein) declines with age in the brain, liver, skeletal muscle and white adipose tissues. SIRT1 expression is age-dependently reduced in microglia	
	As pro-survival protein. Sirt1 ameliorates oxidative stress induced neural cell death and is down-regulated in Parkinson’s disease	[56]
	Sirt1 was down-regulated in post-mortem brain tissue obtained from patients with Parkinson’s disease, Parkinson’s disease with dementia, dementia with Lewy bodies and Alzheimer’s disease,.	
Circadian clock	Sirt1 plays an important role in translating nutritional cues in the brain	[57]
	In the ventromedial hypothalamus, Sirt1 was found to control circadian rodent behavior under specific conditions of light and food restriction, which also extends to effect circadian gene expression of the central clock in the suprachiasmatic nucleus.	
Immunity	Deletion of Sirt1 in hypothalamic Agouti-related peptide-expressing neurons creates a pro-inflammatory environment, with enhanced effector T-cell activity and decreased regulatory T-cell function	[58]
	Hypothalamic inflammation, glial cells activation and learning and memory impairment were alleviated by swimming exercise plus diet control, which was related to the increasing expression of Sirt1	[59]
Psychiatric disorders	Several Sirt1single nucleotide polymorphisms were over-represented in patients with depression and anxiety disorders	[60]
	Sirt1 levels correlates with anxiety and exploratory drive and is mechanistically linked with serotonin levels in the brain	[61]
	Cocaine or morphine administration increases Sirt1 expression in the nucleus accumbens, a brain region that regulates motivation and reward	[62]
